# Application and Prospect of CRISPR/Cas9 Technology in Reversing Drug Resistance of Non-Small Cell Lung Cancer

**DOI:** 10.3389/fphar.2022.900825

**Published:** 2022-05-10

**Authors:** Lu Huang, Zhi Liao, Zhixi Liu, Yan Chen, Tingwenli Huang, Hongtao Xiao

**Affiliations:** ^1^ Department of Clinical Pharmacy, Sichuan Cancer Center, School of Medicine, Sichuan Cancer Hospital and Institute, University of Electronic Science and Technology of China, Chengdu, China; ^2^ Personalized Drug Therapy Key Laboratory of Sichuan Province, Chengdu, China; ^3^ Department of Gynecology and Obstetrics, Sichuan Academy of Medical Sciences and Sichuan Provincial People’s Hospital, Chengdu, China

**Keywords:** non-small cell lung cancer, drug resistance, CRISPR/Cas9, gene editing, TKIs

## Abstract

Cancer drug resistance has always been a major factor affecting the treatment of non-small cell lung cancer, which reduces the quality of life of patients. The clustered regularly interspaced short palindromic repeats/CRISPR associated protein 9 (CRISPR/Cas9) technology, as an efficient and convenient new gene-editing technology, has provided a lot of help to the clinic and accelerated the research of cancer and drug resistance. In this review, we introduce the mechanisms of drug resistance in non-small cell lung cancer (NSCLC), discuss how the CRISPR/Cas9 system can reverse multidrug resistance in NSCLC, and focus on drug resistance gene mutations. To improve the prognosis of NSCLC patients and further improve patients’ quality of life, it is necessary to utilize the CRISPR/Cas9 system in systematic research on cancer drug resistance.

## 1 Introduction

Lung cancer is one of the most common cancers and is the leading cause of cancer death, accounting for approximately 18% of cancer deaths (Sung et al., 2021). Non-small cell lung cancer (NSCLC) is the most common type of lung cancer, accounting for more than 85% of total lung cancer cases, and the World Health Organization (WHO) classifies NSCLC into adenocarcinomas, squamous carcinomas, and large cell carcinomas based on differences in immunohistochemical markers (Mengoli et al., 2018). The high lethality of lung cancer is associated with difficulty in diagnosis, treatment, and poor prognosis (Woodard et al., 2016). The mainstay of treatment for NSCLC is surgery and adjuvant cisplatin-based therapy (Duma et al., 2019), Many challenges remain in the screening and treatment of lung cancer, and mortality is difficult to control. Although chemotherapy can prolong survival to some extent in patients with moderately advanced NSCLC, the overall response rate is only about 30%, the median survival is 8–12 months, and the 1-year survival rate is 30–40% (Reck and Rabe, 2017). The advent of targeted agents has led to improvements in the treatment of NSCLC.

However, the treatment failure in NSCLC is closely related to the phenomenon of acquired drug resistance and multidrug resistance (MDR) in prognosis. For example, in NSCLC patients harboring EGFR gene mutations, the EGFR-TKI class of drugs is the standard first-line treatment, showing disease progression after 9–13 months despite some therapeutic efficacy (Kelly et al., 2015). Tumors with EGFR-TKI resistance mechanisms had EGFR secondary mutations, bypass or downstream pathway activation: such as HER2 amplification, met amplification, FGFR1 activation, PI3K/Akt pathway activation, BRAF mutation, and loss of PTEN expression (Uchibori et al., 2018; Leonetti et al., 2019).

Clustered regularly interspaced short palindromic repeats/CRISPR associated protein 9 (CRISPR/Cas9) technology is the most powerful gene-editing technology after zinc finger nucleases (ZFNs), transcription activator-like effector nucleases (Talens) (Carroll, 2011; Joung and Sander, 2013), with flexible and convenient features, it is inexpensive and has been widely used in biology, microbiology, agriculture, and animal husbandry.

To further investigate the mechanisms of multidrug resistance in NSCLC and improve the prognosis and quality of life of NSCLC patients, we discuss issues related to NSCLC drug resistance by reversing NSCLC multidrug resistance via CRISPR/Cas9, screening drug-resistant targets, and targeting therapies.

## 2 Mechanism of the CRISPR/Cas9 System

CRISPR/Cas, an acquired immune defense system that evolved during long-term evolution in bacteria and archaea to fight invading viruses and foreign DNA, was first identified in 1987 (Ishino et al., 1987) and was later shown to have powerful gene-editing functions.

CRISPR gene sequences are constituted by multiple short and conserved repeats and non-repetitive sequences called spacers, and CAS proteins are a family of endonucleases. There are three main stages in the mechanism of acquired immune protection by CRISPR/Cas9, which are the acquisition of CRISPR spacer sequences, expression of CRISPR genes, and CRISPR interference (Deveau et al., 2010). When a foreign gene first invades a bacterium, CRISPR/Cas9 recognizes the protospacer adjacent motif (PAM), and cuts the DNA sequence adjacent to the PAM as a candidate protospacer from the foreign DNA, inserts downstream of the leader region of the CRISPR sequence, and repairs. When foreign genes re-invade, CRISPR sequences are transcribed to form pre-CRISPR-derived RNA (pre-crRNA) and trans-acting crRNA (tracrRNA), the former of which is sheared by Cas proteins into mature CRISPR derived RNA (crRNA). Subsequently, a complex consisting of pre-crRNA, tracrRNA and cas9 protein allows recognition of the foreign gene and DNA double-strand cleavage.

**FIGURE 1:**
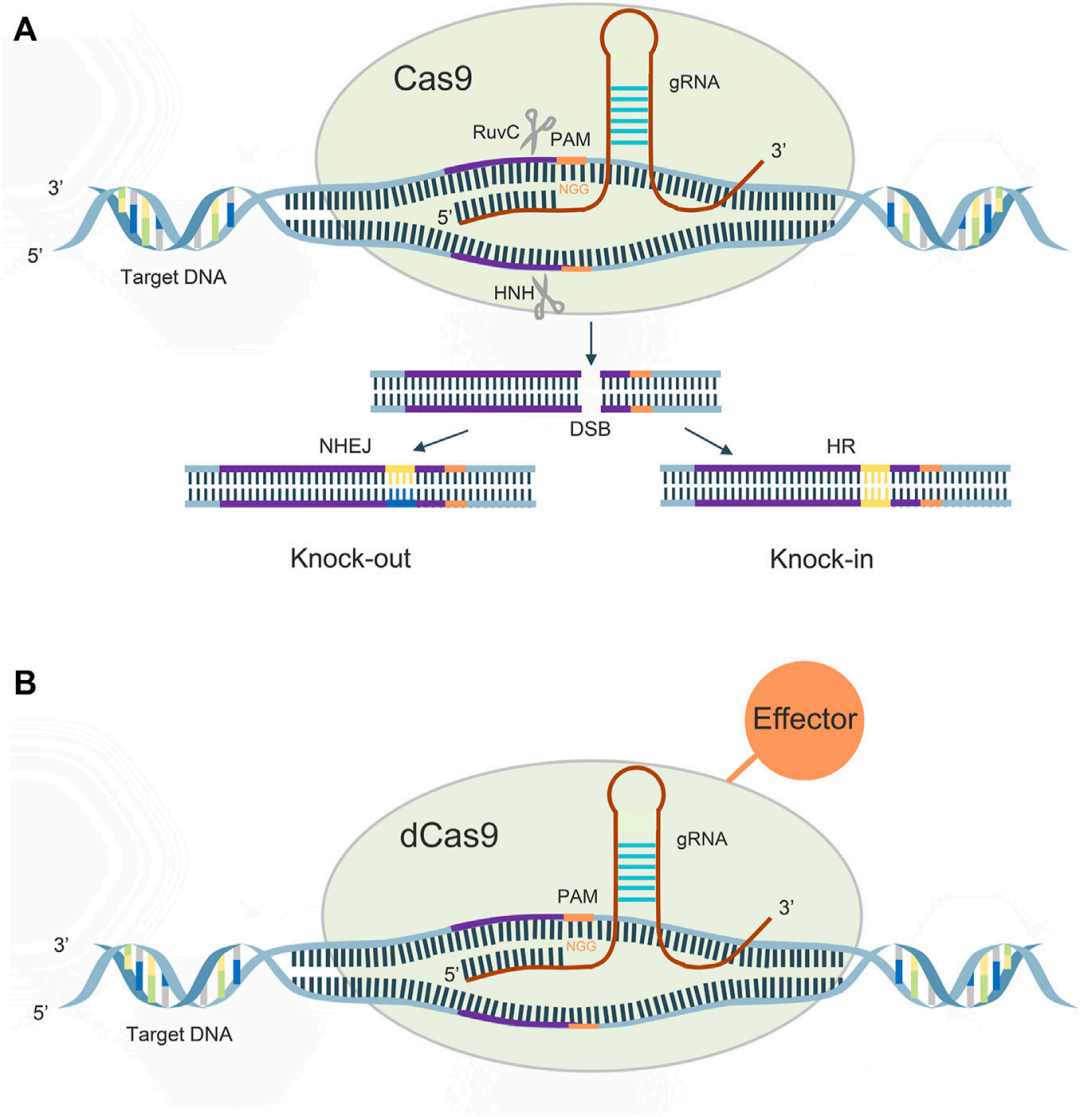
**(A)**: Mechanism of CRISPR/Cas9 system; **(B)**: Mechanism of CRISPR/dCas9 system.

A guide RNA (gRNA), consisting of 20–24 bases, recognizes the PAM on both sides of the target DNA for target sequence cleavage, its HNH enzyme will shear the crRNA complementary DNA strand, while its RUVC active site will shear the noncomplementary strand, causing double-strand breaks (DSBs), and the cell performs DNA repair by non-homologous end joining (NHEJ) and homologous directed recombination (HDR) pathways, thus creating a permanent mutation (Figure 1A).

In 2013, Qi et al. introduced h840a mutations in the HNH domain of the cas9 protein and D10A mutations in the RUVC domain, which rendered the protein activity defective and, although DNA could still be precisely targeted, lost its original function (Qi et al., 2013). The dCas9 can regulate target genes under the guidance of sgRNAs without generating DSBs. The dcas9 protein can carry different effector domains, recruit endogenous transcriptional activators and RNA polymerase to target DNA sequences for target gene activation, and also disrupt transcription factor binding or hinder RNA polymerase binding, thereby silencing target gene expression (McCarty et al., 2020) (Figure 1B).

## 3 Principles of Multidrug Resistance in NSCLC Reversed by CRISPR/Cas9 Technology

**FIGURE 2:**
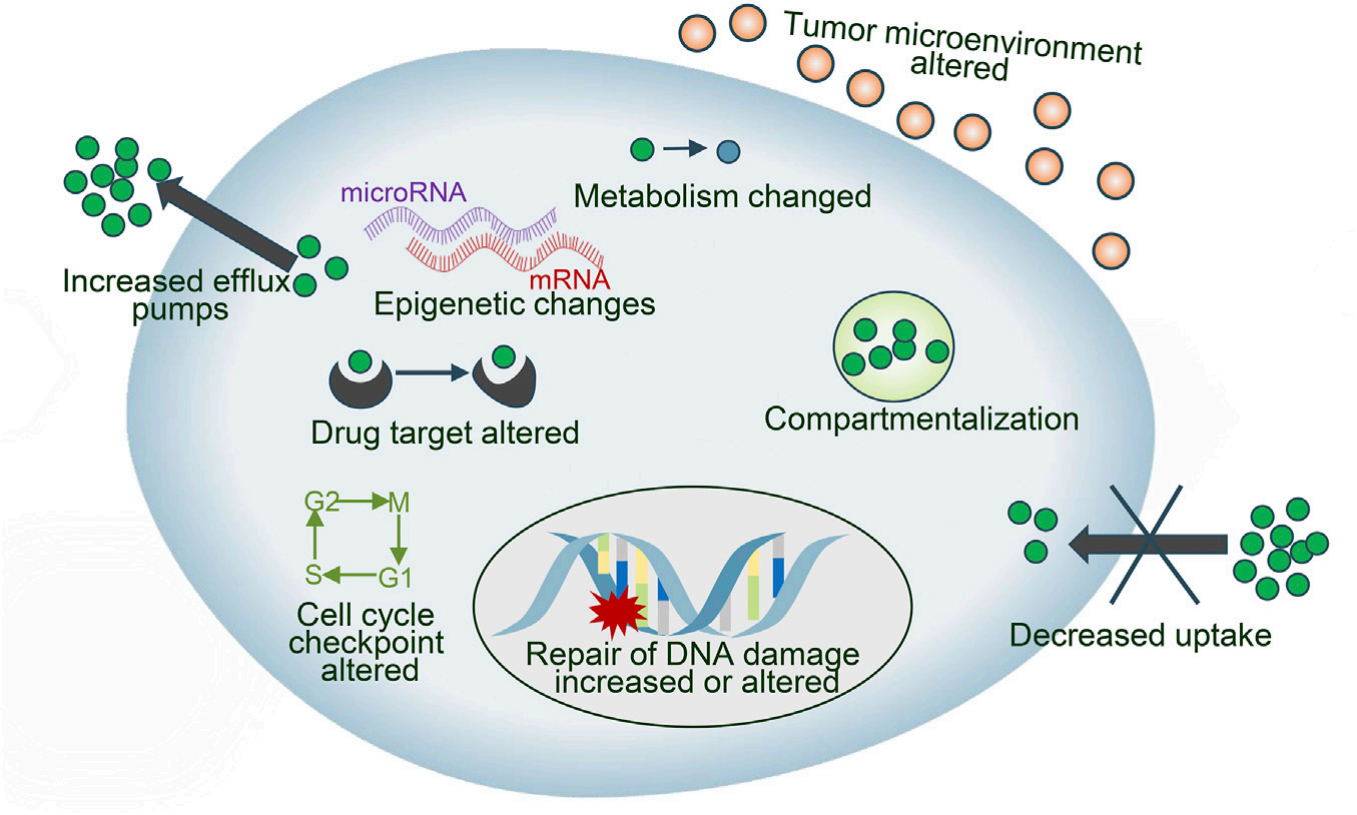
Different mechanisms involved in anticancer drug resistance.

Drug resistance is an essential factor leading to treatment failure in many intractable diseases, which limits the application of chemotherapeutics in NSCLC patients, and the reasons why tumor cells develop drug resistance are complex and variable, mainly including drug inactivation, enhanced drug efflux, epigenetic changes, DNA repair ability, apoptosis inhibition, alteration of drug targets Epithelial-mesenchymal transition (EMT), etc. (Gottesman, 2002; Panda and Biswal, 2019) (Figure 2). These mechanisms can act independently or in combination and act through various signal transduction pathways. CRISPR/Cas9 has been used for the study of drug sensitization and resistance. We discuss the key reasons for drug failure in NSCLC and the role of CRISPR/Cas9 technology.

### 3.1 Studies on Drug Resistance Genes

**FIGURE 3:**
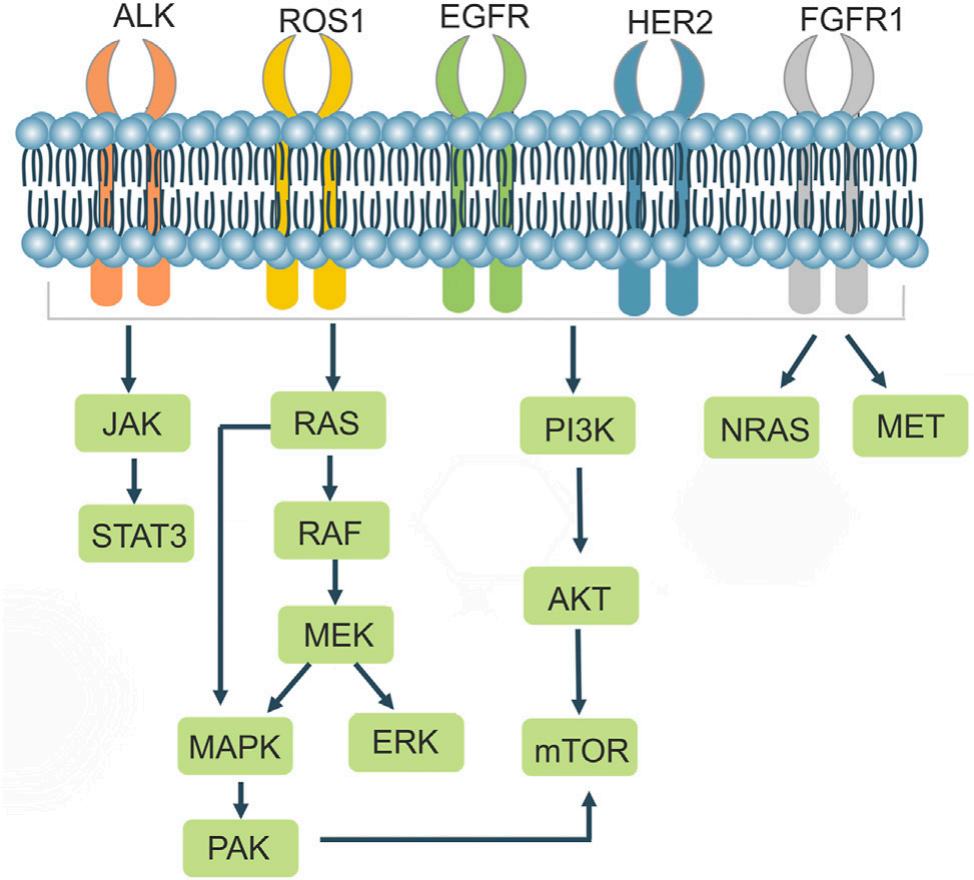
Cell signaling in NSCLC drug resistance.

NSCLCs are strongly associated with mutations in related genes that cause alterations in the structure or number of proteins encoded by the genes, leading to changes in the function of their associated genes. In addition to the well-known EGFR and ALK, BRAF mutations, ros1 rearrangements, RET rearrangements, and others are common in NSCLC. There are a variety of targeted drugs acting on the relevant receptors (Table 1; Figure 3). The CRISPR/Cas9 system can be used to remove the functional regions of drug resistance genes, thereby reversing drug resistance. It can also be used to knock out or overexpress drug resistance genes in cell lines and animals, making it easier to research drug resistance mechanisms in tumors.

**TABLE 1:** Summary of targeted therapeutic drugs for non-small cell lung cancer.

Targrt	Mechanism of Action	Drug	Usage	Ref
First generation EGFR-TKIs	Inhibits by binding to the ATP site of the EGFR receptor	Erlotinib	For first-line treatment of locally advanced or metastatic NSCLC with sensitive gene mutations in EGFR.	Zhou et al. (2011)
		Gefitinib	It is used for single drug continuous treatment of locally advanced or metastatic NSCLC with platinum and docetaxel chemotherapy failure	Goss et al. (2013)
Second generation EGFR-TKIs	Blocking the EGFR-HER2 signaling pathway	Afatinib	It can significantly improve the progression free survival, objective response rate (ORR) and 8-weeks disease control rate	Park et al. (2016)
Third generation EGFR-TKIs	Play a role in secondary drug resistance. the binding of ALK	Osimertinib	Targeted treatment of patients with EGFR mutation and T790M drug resistance mutation significantly prolonged PFS in patients with NSCLC.	Cho et al. (2019)
First generation ALK-TKIs	Competitive binding to ATP binding sites blocks. The binding of the ALK enzyme to ATP, hinders the subsequent autophosphorylation process, and leads to the inactivation of the ALK downstream signal pathway	Crizotinib	It can effectively inhibit NSCLC caused by ROS 1 gene rearrangement	Moro-Sibilot et al. (2019)
Second generation ALKK-TKIs		Ceritinib	It is applicable to NSCLC patients who progress after treatment with kezotinib or cannot tolerate its toxic and side effects	Soria et al. (2017)
BRAF inhibitor	The continuous activation of BRAF gene leads to the over activation of MEK/ERK signaling pathway, which leads to tumor production and even tumor metastasis	Dabrafenib	Combined with trimetinib to treat patients with advanced NSCLC with braf-v600e mutation	Planchard et al. (2017)
c-Met inhibitor	c-MET can affect the downstream PI3K/Akt and MAPK pathways, and abnormal c-met activity leads to abnormal metabolism	Tivantinib	Combined with EGFR-TKI can effectively prolong the PFS of EGFR mutant NSCLC.	Yoshioka et al. (2015)

#### 3.1.1 EGFR

EGFR is a tyrosine kinase receptor that Homo*—*or heterodimerizes with ligands to cause autophosphorylation, which in turn regulates downstream signaling pathways leading to tumor proliferation, invasion, metastasis and angiogenesis. Mutations in the EGFR kinase domain are present in approximately 10%–40% of patients with NSCLC. Treatment of EGFR mutant lung cancers with EGFR-TKIs effectively inhibits tumor progression and prolongs progression free survival (PFS) in patients with NSCLC compared with standard chemotherapeutic agents (Schrank et al., 2018). Approximately 90% of EGFR mutations are caused by mutations in exon 19 (exon 19 deletion mutation) and exon 21 (L858R) (Camidge et al., 2014; Robichaux et al., 2018). After administration of TKIs, most patients develop acquired resistance, which is usually caused by a secondary mutation at position 790 in exon 20 (Cross et al., 2014), Targeting the third-generation EFGR-TKI Osimertinib for this resistance occurs where the EGFR c797s mutation blocks efficient binding of Osimertinib to the target EGFR c797 site (Jia et al., 2016). Tang et al. proposed an individualized molecular surgical treatment strategy for EGFR mutant lung cancer using CRISPR/Cas9 technology, which generates breaks at mutation sites or exons. The wild-type sequence containing the exon and the donor DNA with its left and right homology arms would then replace the mutated sequence or exon by HDR, an approach that would eradicate the drug resistance gene and thus prevent cancer progression (Tang and Shrager, 2016). Liu et al. used CRISPR/Cas9 technology to create renal cell carcinoma (RCC) cell lines with EGFR knockout, which significantly inhibited cancer cell proliferation and induced cell arrest in the G2/M phase. However, knocking out EGFR resulted in high ERK expression, but the authors discovered that ERK and Akt could be inhibited by Sunitinib (a multi-targeted TKI) in combination (Liu et al., 2020), suggesting that CRISPR mediated knockout of drug resistance genes may be a promising option for future disease treatment.

#### 3.1.2 ALK

Anaplastic lymphoma kinase (ALK), which belongs to the insulin receptor (IR) superfamily, is a highly conserved receptor tyrosine kinase. ALK rearrangements are found in approximately 3%–7% of NSCLC patients (Devarakonda et al., 2015). ALK is mutated as a fusion with echinoderm microtubule-associated protein like 4 (EML4), encoding the form of an EML4-ALK fusion protein that leads to ALK dimerization, which results in the activation of ALK and its downstream signaling pathways such as JAKs/STAT3 and RAS/MEK/ERK, leading to aberrant cell proliferation and differentiation and promoting tumorigenesis (Soda et al., 2007; Sasaki et al., 2010). In 2014, Blasco et al. designed sgRNAs targeting intron 14 of the EML4 gene and intron 19 of the ALK gene in mice, generated DSBs using Cas9, and generated EML4-ALK rearrangements in non-small cell lung cancer cells, which were able to promote tumor formation in the lungs of mice, demonstrating the importance of the CRISPR/Cas9 system for studying chromosomal rearrangements (Blasco et al., 2014). EML4-ALK exhibits potent oncogenic properties both *in vitro* and *in vivo*, in which tumor development can be rapidly suppressed using ALK TKIs (Soda et al., 2008). Tumor cells often develop acquired resistance to ALK inhibitors, resulting from secondary mutations in the patient’s kinase domain, gene amplification, and activation of alternative signaling pathways (e.g., EGFR, kit, IGF1R, etc.) and epithelial mesenchymal transformation (Spaans and Goss, 2014; Kong et al., 2019). ALK creates secondary mutations that promote an altered spatial conformation of the kinase, weaker binding to the drug, or stronger binding to ATP, leading to the development of drug resistance. The initial ALK mutation was the L1196m mutation, and the leucine residue L1196 in the ALK kinase domain, located at the bottom of the ATP binding pocket, is mutated to methionine. The thioether side chain of methionine would create a steric hindrance to hinder the binding of the ALK-TKI Crizotinib to the ALK kinase, resulting in Crizotinib resistance (Doebele et al., 2012).

#### 3.1.3 ROS1

The receptor tyrosine kinase ROS proto-oncogene 1 (ROS1) belongs to a group of receptor tyrosine kinases in the insulin family of receptors, and ROS1 rearrangements are observed in approximately 1%–2% of patients with NSCLC (Gainor and Shaw, 2013). The kinase domains of ALK and ROS1 share homology, and Crizotinib, an ALK-EML 4 inhibitor, was used to interfere with ROS1 fusion gene-positive and ALK-EML4 fusion gene-positive lung cancer cells, and Crizotinib was found to inhibit the growth of hcc78 cells (ROS1 fusion gene-positive) (Bergethon et al., 2012), Accordingly, some ALK-TKIs have been shown to be effective in patients with ros1 rearrangement (Huber et al., 2014). Choi et al. achieved the first CD74-ROS1 translocation event utilizing CRISPR/Cas9 technology in 2014, suggesting that Cas9-induced DSB can result in chromosome translocation (Choi and Meyerson, 2014). Sato et al. designed gRNAs to target EZR intron 9 and ROS1 intron 33 and successfully generated EZR/ROS1 fusions in HBECp53 lung adenocarcinoma cells, which highly induced the phosphorylation of MEK and ERK, and the MEK/ERK signaling pathway can mediate the primary or acquired resistance to ROS11 TKIs in ROS1 rearranged lung adenocarcinoma patients. Using a combination of Selumetinib and Crizotinib, the authors effectively inhibited the growth of ros1 fusion positive cells *in vitro* and *in vivo* (Sato et al., 2020), providing a therapeutic strategy for NSCLC.

#### 3.1.4 KRAS

Kirsten rat sarcoma viral oncogene (KRAS) belongs to the RAS protein family, and KRAS mutation is a common type of mutation in non-small cell lung cancer. When KRAS is bound to guanosine triphosphate (GTP), it is activated and can activate downstream BRAF/MEK/ERK and PI3K/Akt/mTOR signaling pathways (Friedlaender et al., 2020). The mutation rate of KRAS in NSCLC is 20%–30%, and about 97% of these mutations are point mutations in codon 12 or 13 in exon 2 (Rotow and Bivona, 2017). KRAS is a marker of resistance to EGFR-TKIs drugs, and KRAS mutation is an indicator of poor prognosis in NSCLC. So far, no effective KRAS inhibitors have been developed. Gao et al. used the CRISPR/Cas9 system to knock out KRAS G12S, used dcas9 KRAB to bind to the target. KRAB, a transcription inhibitor, can downregulate mRNA transcription. Cas9-sgG12S suppressed the proliferation of tumor cells by inhibiting the production of the KRAS (G12S) protein in A549 cells, as well as the phosphorylation levels of downstream molecules Akt and ERK. The tumor volume reduced by 46%, the tumor volume decreased by 30%, and the expression of KRAS mutant protein decreased dramatically in A549 mice treated with Adv-Cas9-sgG12S (Gao et al., 2020).

#### 3.1.5 BRAF

V-Raf mouse sarcoma viral oncogene homolog B (BRAF) is a serine/threonine kinase that is downstream of KRAS in the MAPK signaling cascade pathway. BRAF is mutated in 60% of melanomas and drives oncogenes for a variety of malignancies such as colorectal, ovarian, and papillary thyroid cancer. RAS-GTP binding to the receptor-binding domain (RBD) activates RAF, leading to RAF phosphorylation and the induction of MEK and ERK activation, which results in cell proliferation and differentiation (Wan et al., 2004). Ding et al. tested the amplification refractory mutation system in 1680 NSCLC patients and found that the BRAF mutation rate was 1.7%, and was mostly found in lung adenocarcinoma patients and female patients (Ding et al., 2017). The predominant type of mutation in the brae gene in NSCLC is V600E, with a mutation rate of over 50% (Li et al., 2014), and investigators have found two mutations, V458L and K438T, on exon 11 in lung adenocarcinoma (Brose et al., 2002). Resistance to the BRAF inhibitor dabrafenib often develops within 8 months (Flaherty et al., 2010; Chapman et al., 2011). As BRAF mutations are more common in melanoma, studies utilizing CRISPR/Cas9 for chemotherapeutic agents have often revolved around the melanoma. Wu et al. developed a light-inducible CRISPR/Cas9 system to cleave the mutated BRAF gene (BRAF V600E), which promotes melanoma cell apoptosis and effectively inhibits melanoma cell proliferation, invasion, and migration (Wu et al., 2020).

#### 3.1.6 MET

C-MET proto-oncogene, receptor tyrosine kinase (c-MET), a transmembrane receptor encoded by the met gene, belongs to the hepatocyte growth factor (HGF) receptor family, and HGF, in combination with c-MET, undergoes phosphorylation and autophosphorylation and activates downstream PI3 K/Akt and MAPK signaling pathways (Pasquini and Giaccone, 2018), MET amplification accounts for 5%–20% of NSCLC patients and is a poor prognostic factor for EGFR-TKI acquired resistance (Bubendorf et al., 2017). Met exon 14 mutations are common and account for 3% of lung adenocarcinomas (Schrock et al., 2016). Crizotinib acts as a tyrosine kinase receptor inhibitor capable of inhibiting c-MET. Togashi and others used CRISPR/Cas9 system to knock out the exon of MET 14 in HEK293 cell line, MET phosphorylation raised, protein expression increased, cell proliferation was reinforced, and cell sensitivity to Crizotinib was improved (Togashi et al., 2015), demonstrating that targeted therapy for MET exon 14 deleted non-small cell lung cancer holds promise.

#### 3.1.7 Other Genes

Nonspecific conventional chemotherapy drugs, such as cisplatin, paclitaxel, and etoposide, are also commonly used in the treatment of NSCLC. Chen et al. silenced Rsf-1 in NSCLC by CRISPR/Cas9, which inhibited cancer cell inhibition and migration and promoted cancer cell apoptosis, demonstrating that Rsf-1 regulates NF-κ B pathways to influence NSCLC sensitivity to paclitaxel (Chen et al., 2017). Aurora-B is a key factor regulating mitosis and is frequently overexpressed in lung cancer. Yu et al. knocked down Aurora-B in the A549 cell line by CRISPR/Cas9 technology, and demonstrated that Aurora-B could confer NSCLC drug resistance by inhibiting cell proliferation, p53 related DNA damage response and apoptotic pathways, while knocking down Aurora-B was able to restore cell sensitivity to cisplatin and paclitaxel (Yu et al., 2018). Zhang et al. found that transducing-like (β) receptor 1 (tbl1xr1) was overexpressed in NSCLC and promoted cancer progression by regulating the MEK and Akt signaling pathways through its master regulator c-MET, knockdown of tbl1xr1 by CRISPR/Cas9 in A549 and H460 cell lines resulted in an increase in the number of cells in G0/G1 phase, inhibited cell proliferation and migration, and promoted apoptosis with a concomitant increase in sensitivity to cisplatin (Zhang T. et al., 2020).

### 3.2 Application of CRISPR/Cas9 in Screening Drug Resistance Genes

CRISPR/Cas9 technology is also being used for genetic screening of potential drug resistance in NSCLC. Previously, RNA interference (RNAi) - based genetic screens, the mainstay of genome-wide loss of function screens, have been effective in identifying genes in tumor cells that can respond to chemotherapeutic agents and in studying signaling pathways. However, there are a series of challenges in the application of RNAi. For example, the high specificity of RNAi is relative, in some cases siRNAs produce the off-target phenomenon. RNAi cannot wholly block the expression of genes in mammalian cells, especially those that are abnormally highly expressed (Jackson et al., 2006; Mullenders and Bernards, 2009). CRISPR/Cas9 technology can activate or repress gene expression and can label functional regions at specific genomic loci, resulting in accurate genome editing with the advantages of fewer false positives and lower off-target effects (Evers et al., 2016), which has now been applied to screen drug resistance genes in a variety of tumors.

Zeng et al., through genome-wide CRISPR/Cas9 gene screening, found that inactivation of GPCR related effectors produced obvious synergistic effects with EGFR inhibition in EGFR mutated NSCLC cells, deficiency of GPCR related effector - RIC8A could improve cell sensitivity to chemotherapeutic drugs, and targeting RIC8A is promising as a new approach to preventing EGFR-TKI resistance in NSCLC (Zeng et al., 2019). Lee et al. used CRISPR/Cas9 libraries to screen human lung cancer cell lines (NCI-H820) and knockdown of the genes MDM4, PSMA6, PSMB6, ANAPC5, and CDK1 increased the sensitivity of lung cancer cells to the EGFR-TKI Erlotinib, the MDM4 inhibitor nutlin-3 synergized with PSMA6, and the PSMB6 inhibitor Carfilzomib synergized with Erlotinib *in vitro* cell lines and *in vivo* patient-derived xenograft experiments, can promote tumor cell death, target cell cycle or protein ubiquitination pathways, and may inhibit Erlotinib resistance progression (Lee et al., 2021).

### 3.3 Modification of Cellular Transport Pathways

Cancer cells often efflux chemotherapeutic agents out of the cell to lower intracellular drug concentrations by up regulating one or more adenosine triphosphate binding cassette (ABC) membrane transporters (Mollazadeh et al., 2018). Three transporters multidrug resistance protein 1 (MDR1), multidrug resistance-associated protein 1 (MRP1), and breast cancer resistance protein (BCRP) - have been implicated in cancer resistance (Sakaeda et al., 2002; Cole, 2014; Mao and Unadkat, 2015). P-glycoprotein (P-gp), a member of the ABC superfamily of structural transporters that have been extensively studied, is encoded by MDR1 (Panczyk et al., 2007), is widely distributed in tissues such as the brain, lung, liver, kidney and gastrointestinal tract (Gupta et al., 2015), and is highly expressed within tumor cells, which confers drug resistance (Ambudkar, 1995; Li et al., 2016). Studies in many different types of cancer have shown that increased expression of any one of these transporters in cancer cells leads to suboptimal clinical outcomes. Jia et al. showed that the expression level of P-gp in ovarian cancer tissues was significantly higher than that in adjacent normal tissues, and increased with higher clinical stage of ovarian cancer (Jia et al., 2018). EL-Masry et al. demonstrated that in adult acute myeloid leukemia (AML) patients, BCRP was highly expressed in 34 out of 50 adult AML patients (68%) (El-Masry et al., 2018). In chronic myeloid leukemia (CML), tumor sensitivity can be increased using febuxostat, a BCRP inhibitor (Ito et al., 2021).

Using the CRISPR/Cas9 system to target the MDR1 gene in the MDR cell lines KBV 200 and HCT-8/V, Yang et al. were able to improve vincristine and doxorubicin sensitivity in MDR cancer cells (Yang et al., 2016a). The PI3K inhibitor BAY-1082439 was able to down regulate P-gp and BCRP expression, and nonviral transgenic vector-mediated CRISPR/Cas9 knockdown of PI3K in non-small cell lung cancer H460 cell line and its resistant subline H460/MX20 110 α And 110.0 β Subunit, leading to downregulation of P-gp and BCRP and reversing P-gp-mediated drug resistance (Zhang L. et al., 2020).

### 3.5 CRISPR/Cas9 for Epigenetic Regulation

Epigenetic regulation of cancer cells has an important role in the process of drug resistance. Epigenetics refers to the regulatory mechanisms of gene expression that result in an altered phenotype through the modification of DNA bases. Many of the genes that play a key role in the process of cancer drug resistance often have abnormal alterations in epigenetics to escape the body’s immune surveillance. Many of the sites that are mutated at high frequency on the drug-resistant genomes of tumors are genes encoding enzymes associated with epigenetic regulation (Yu et al., 2011; Azad et al., 2013). Common epigenetic regulations include DNA methylation, histone modification, noncoding RNA regulation, and chromatin remodeling, among others (Dawson and Kouzarides, 2012). DNA methylation is the addition of a methyl group to the cytosine of certain specific regions (i.e., the Cp G Islands) where methylation occurs, leading to the expression of the gene being affected. Transcriptional inactivation, silencing of tumor suppressor genes when aberrantly methylated, or activation of oncogenes due to DNA hypomethylation may underlie tumorigenesis and chemotherapeutic resistance (Liu B. et al., 2016). Terai et al. showed that gefitinib-resistant lung cancer cells had significantly increased methylation relative to parental cells (Terai et al., 2015). Protein modification refers to the process by which histones undergo methylation, acetylation, phosphorylation, ubiquitination and other modifications under the action of related enzymes (Audia and Campbell, 2016). In hepatocellular carcinoma (HCC), G9a, a histone methyltransferase, promotes HCC proliferation and metastasis by regulating the dimethylation level of rarres3 histone (Wei et al., 2017). In 2016, Okano et al. initially demonstrated the essential role of the dCas9-Tet1 and dCas9-Dnmt3a systems for epigenetic regulation by using Tet1 and Dnmt3a catalytically inactive cas9 fusion proteins to target the brain-derived neurotrophic factor (BDNF) promoter Ⅳ and distal enhancer of myogenic determination factor (MyoD) (Liu X. S. et al., 2016). In terms of histone deacetylation modification, Liu et al. fused dCas9 to HDAC1 and achieved deacetylation of histones at the KRAS promoter and effectively silenced the oncogene KRAS, providing a novel approach for cancer therapy (Liu et al., 2021).

Rakshit et al. used CRISPR/Cas9 to knock down BRCA1 in human CD4 + T helper cells and demonstrated that the expression of the BRCA1 gene in the VEGFA and aimp1 loci was suppressed in NSCLC, and aberrant expression of multiple DNA damage/repair factors was found in the aimp1 and VEGFA loci. However, knockdown of BRCA1 results in high levels of R-loop formation at the VEGFA and AIMP1 loci, and the R-loop structure is one of the major intracellular causes of genomic instability (Rakshit et al., 2021). Choudhury et al. used the CRISPR/dCas9 system at the promoter region of BRCA1 to reduce DNA methylation and reactivate gene expression to restore function to BRCA1 for the purpose of cancer suppression (Choudhury et al., 2016). Kang et al. used CRISPR/Cas9 to change the CpG dinucleotides in the promoter region to unmethylated dinucleotides and achieved selective DNA demethylation by targeting methylated CpG sites using the CRISPR/dCas9-Tet1 system (Kang et al., 2019).

### Studies on miRNA Expression

MicroRNAs (miRNAs), a class of endogenous non-coding RNAs with 19–24 nucleotides in length, play key roles in regulating tumor cell proliferation, differentiation, migration, invasion, and miRNAs and their mediated signaling pathways are directly involved in the regulation of multiple cell biological pathways and cisplatin response in non-small cell lung cancer (Zang et al., 2017; Santos and Almeida, 2020). Yang et al. demonstrated that miR-26a could inhibit the HMGA2 mediated E2F1-Akt signaling pathway by down regulating intracellular high mobility group a 2 (HMGA2) expression, which in turn enhanced cisplatin resistance (Yang et al., 2016b). MiRNAs can regulate non-small cell lung cancer apoptosis, and then regulate the drug resistance of cells. Qiu et al. found that miR-503 specifically targeted anti-apoptotic protein Bcl-2, and then reversed cisplatin resistance in non-small cell lung cancer (Qiu et al., 2013).

Overexpression of miR-421 in NSCLC promoted lung cancer cell migration and invasion and increased the resistance of lung cancer cells to paclitaxel. CRISPR/Cas9 knockout β- Catenin downregulates miR-421 levels in A549 cells (Duan et al., 2019). Knockdown of LHX6 in HCC827/ER cells by CRISPR/Cas9 system reversed the reduced cell invasion and Erlotinib resistance caused by downregulation of miR-214 (Liao et al., 2017). Overexpression of miR-1304 significantly decreased the number of NSCLC cells and promoted apoptosis. Li et al. showed that the expression of HO-1 was significantly increased by knockdown of endogenous miR-1304 by CRISPR/Cas9, and miR-1304 inhibited NSCLC cell growth by targeting HO-1, demonstrating that modulation of miR-1304/HO-1 may be a novel therapeutic avenue (Li et al., 2017).

### 3.6 Studies on Epithelial Mesenchymal Transition

Epithelial to mesenchymal transition (EMT) refers to the process in which, under certain conditions, cells of the epithelial phenotype appear to have downregulated expression of characteristic proteins of the epithelial phenotype, whereas cells of the mesenchymal phenotype are upregulated, that is, epithelial cells undergo a morphological transition to a fibroblastic or mesenchymal phenotype, and cells undergo loss of cell polarity, which enables increased motility (Tsai and Yang, 2013). Key signaling pathways involved in EMT include TGF-β, Wnt, Notch and Hedgehog et al. (Gonzalez and Medici, 2014; David et al., 2016; De Francesco et al., 2018; Teeuwssen and Fodde, 2019). Several methodologies have been utilized to investigate the role of various genes in the EMT process in various diseases. RNAi is often used in EMT research, but because its low specificity is inevitable, CRISPR/Cas9 is now being widely used to help us identify potential therapeutic targets for EMT-associated diseases. For example, Survivin, one of the main members of the inhibitor of apoptosis (IAP), was highly expressed in a variety of tumor tissues and cells and promoted EMT, which was associated with proliferation, migration and chemoresistance in various cancers, such as breast cancer, non-small cell lung cancer, and prostate cancer. Using the CRISPR/Cas9 system, Zhao et al. showed that TGF-β could be attenuated by knockdown of BIRC5, the gene encoding Survivin, in ovarian cancer cells SKOV3 and OVCAR3 signaling that inhibits cancer cell proliferation and migration and restores sensitivity to paclitaxel (Zhao et al., 2017).

Using CRISPR/Cas9 mediated silencing of Smad3/Smad4, Tong et al. showed decreased mRNA expression of Myocardin (MYOCD) and downregulation of TGF-β Induced invasion and epithelial-mesenchymal transition of non-small cell lung cancer cells (Tong et al., 2020). Perumal et al. used the CRISPR/Cas9 system to knock out the phosphatase and tensin homolog (PTEN) in the non-small cell lung cancer cell lines A549 and NCI-H460 by Nuclear translocation of β-catenin and Snail/Slug in lung cancer cells promotes EMT, which leads to metastasis (Perumal et al., 2019). Mesenchymal cells are poorly sensitive to EGFR inhibitors. Raoof et al. identified FGFR1 as the highest genomic target to re-sensitize cells to EGF816 using a genome-wide CRISPR screen, and EGFR inhibitors synergize with FGFR1 inhibitors to overcome chemoresistance in NSCLC with mesenchymal features (Raoof et al., 2019).

## 4 Delivery Method of CRISPR/Cas9 System

### 4.1 Physical Methods

In *in vitro* experiments, physical methods are often used to deliver the CRISPR system, which is a simple and efficient way, mainly including electroporation and microinjection. Cas9-sgRNA complex encoded by plasmid is delivered through the cell membrane. Microinjection has high costs and low efficiency. Chen et al. used electroporation to efficiently deliver cas9/sgRNA ribonucleoprotein to mouse fertilized eggs to realize mouse genome editing (Chen et al., 2016).

### 4.2 Nonviral Vector

Nonviral vectors are less immunogenic, have larger capacities, and can deliver large genes but less efficiently. Lipid nanoparticles (LNPS) are one of the most commonly used nucleic acid delivery systems. Negatively charged nucleic acids complex with positively charged lipids via electrostatic interactions to form lipid nanoparticles, which can protect nucleic acids from destruction by nucleases and enter target cells via endocytosis (Chen et al., 2020). The method is safe, cost-effective and straightforward, but has low delivery efficiency. Cationic liposomes, zwitterionic liposomes, and liposome-like materials have been used in CRISPR delivery systems. Zhang et al. constructed a novel delivery system based on polyethene glycol phospholipid modified cationic lipid nanoparticles (PLNP), which significantly downregulated Polo-like kinase 1 (PLK-1) protein and inhibited melanoma growth *in vivo* and *in vitro* (Zhang et al., 2017).

Polymeric carriers are widely used for gene-drug delivery with the advantages of easy synthesis, safety and no immunogenicity. Kang et al., using polymer derived Cas9 complexed with sgRNA targeting antibiotic resistance by covalently modifying the protein with a cationic polymer to induce DNA double-strand breaks, demonstrated potential applications compared to liposomes for enhanced delivery efficiency (Kang et al., 2017).

Inorganic nanoparticles can also be used to deliver nucleic acids with the advantages of low toxicity, high stability, flexibility and easy regulation (Duncan et al., 2010). Mout et al. used arginine functionalized gold nanoparticles (ArgNPs) to cotransport cas9 protein and sgRNA, and ArgNPs delivered RNP to both the cytoplasm and nucleus and achieved 90–95% delivery efficiency (Mout and Rotello, 2017).

### 4.3 Viral Vectors

Lentiviral (LVs) vectors, based on the HIV-1 virus and consisting of a spherical structure composed of single-stranded RNA, have been widely used to deliver CRISPR/cas9. The major advantage of lentiviral vectors is that they can reach 7 kb in load and accommodate the SpCas9 gene and one or more sgRNAs. Holmgaard et al. delivered CRISPR/Cas9 system based on lentiviral vectors. Knockdown of the vascular endothelial growth factor A (Vegfa) gene has led to new treatments for ocular diseases (Holmgaard et al., 2017).

Adenoviruses (ADVs) are non enveloped linear double-stranded DNA viruses with a wide host range, genetic stability, high transduction efficiency and large loading capacity. Jin et al. used Gateway cloning technology to develop an integrated adenoviral vector without traditional enzymatic digestion and ligation, improving transduction efficiency (Jin et al., 2019).

Adeno associated virus (AAVs) is extremely low immunogenic relative to other viral vectors and has safety and therapeutic potential. AAV sequences are long-lived in non-dividing cells, provide stable transgene expression, and are the most widely used viral vector to deliver CRISPR/Cas9 systems.

In 2021, Zhang Feng’s team developed a new delivery vector-selective endogenous encapsidation for cellular delivery (SEND), which is composed of a retrovirus-like protein, PEG10, that binds to its mRNA and forms vesicles around it. The research team modified and designed it to package and deliver specific RNAs (Segel et al., 2021).

## 5 Deficiencies and Challenges

The CRISPR/Cas9 system can well break through the limitations of traditional diagnosis and combat tumor resistance, and is a promising therapy, but some problems still need to be solved.

The off-target effect of CRISPR/Cas9 system is a widespread phenomenon, and the serious consequences caused by off-target limited CRISPR/Cas9 system from basic research to clinic, mainly due to the local matching between the recognition sequence of sgRNA and non-target DNA, the structure of sgRNA, PAM sequence The cas9 protein, along with regulatory small molecules of the DSB pathway, among others, all contribute to targeting efficiency (Zhang et al., 2015). When the concentration of the cas9 sgRNA complex is increased, the specificity of cas9 cleavage is reduced and the RNA polymerase II transcription system can be used to express sgRNA and control the amount of sgRNA expressed (Kiani et al., 2014). In addition to guiding cas9 to bind to specific targets, sgRNAs can also affect the specificity of targets (Pattanayak et al., 2013). Increasing the guide sequence length of the sgRNA did not improve target specificity, which was found to be increased when the sgRNA contained 17–18 nucleotides (Fu et al., 2014). To improve the specificity of DNA cleavage, investigators have used mutant dCas9 that forms a dimer with the nuclease Fok I (FOK I-dCas9) to reduce off-target effects, which is more than 140 fold more specific than wild-type cas9 (Guilinger et al., 2014). Meanwhile, direct delivery of purified recombinant cas9 protein and sgRNA into cells can also reduce off-target effects (Kim et al., 2014).

Recent studies have found that the CRISPR/Cas system may adversely affect cell growth, and Leibowitz et al. found that CRISPR/Cas9 genome editing induces structural changes in the nucleus, micronuclei, and chromosomal bridges, leading to the occurrence of chromosomal rearrangement processes (Leibowitz et al., 2021). Delivery vectors for CRISPR/Cas9 are closely related to gene editing efficiency, and it is crucial to find safe, efficient, and specific vectors. The loading capacity of vectors is limited, and it is challenging to load Cas9 and gRNA into a certain size carrier and improve the delivery efficiency *in vivo*. Currently, the most widely used *in vivo* experiments are viral vectors, but some nonviral vectors still need to be developed for more safe and effective delivery tools (Chen et al., 2020). The intein-mediated split-Cas9 system, which reconstitutes a full-length SpCas9 protein by fusing the segmented two segments of SpCas9 with the N-terminus of intein fused to the C-terminus, respectively, and mediates CIS splicing when both fusion proteins are coexpressed, has been shown to be effective in addressing the challenge of insufficient AAV loading capacity (Truong et al., 2015). Carlson-Stevermer et al. used short RNA and streptavidin to assemble and deliver a CRISPR repair kit to DNA cleavage sites, greatly improving the precision of gene editing, which resulted in an 18 fold increase in accuracy compared with conventional CRISPR Technology (Carlson-Stevermer et al., 2017). At the same time, there is a certain risk of pathogenicity associated with viral vectors, and safety concerns are also issues to consider when viral vectors are used in animal experiments.

At the same time, gene knockout causes permanent changes in genetic material and there are hidden dangers of mutation. Therefore, it is necessary to find new methods to solve this problem. Prime editor is a more accurate gene-editing method. Its protease is fused by cas9 notch enzyme (h840a) and reverse transcriptase. It can accurately insert and delete the target site without introducing DSB and donor DNA templates. Compared with HDR, it has higher efficiency, fewer by-products and a lower off-target rate (Anzalone et al., 2019).

P53 is a tumor suppressor gene, and CRISPR/Cas9 can induce p53 mediated DNA damage response, resulting in cell cycle arrest and other phenomena and reducing the efficiency of genome editing. Whereas inhibition of p53 predisposes cells to the effects of other oncogenic mutations (Haapaniemi et al., 2018; Jiang et al., 2022). Therefore, it is necessary to monitor the function of p53 when CRISPR/Cas9 is used clinically.

There was a study that detected antibodies against Sacas9 and Spcas9 in 78% and 58% of donor sera, respectively. Anti-Sacas9 T cells and anti-Spcas9 T cells were found in 78% and 67% of donors, indicating that there is human immunity to cas9 protein (Charlesworth et al., 2019). In the future, we need more research to determine the safety and effectiveness of CRISPR/Cas9 system.

## 6 Conclusion

CRISPR/Cas9 gene-editing technology has developed rapidly since its inception. Compared with ZFNs and TALENs, CRISPR/Cas9 gene-editing technology is more straightforward and efficient. It is suitable for ordinary laboratories and greatly promotes the progress of life science and basic medical research. Now there is a genome-wide targeted CRISPR/Cas9 system, which contains all genes of mouse embryonic stem cells and human cells (Wang et al., 2014). Lu et al. carried out the world’s first human clinical trial based on CRISPR/Cas9 gene-editing technology. Immune cells were extracted from the blood of a patient with metastatic non-small cell lung cancer. The PD-1 gene that inhibits immune function was knocked out *in vitro* by CRISPR technology, and then amplified and reinfused into the patient’s body to achieve the effect of anti-tumor. The safety and feasibility of this therapy in NSCLC were proved for the first time (Lu et al., 2020). In addition, several laboratories are also competing to plan clinical trials. Researchers at the University of Pennsylvania have launched trials on myeloma, sarcoma and melanoma.

The use of CRISPR/Cas9 gene-editing technology has also caused ethical and social problems. Due to the disadvantages such as being off-target, CRISPR/Cas9 gene-editing technology may cause some additional harm to patients, and the potential high risk does not allow CRISPR/Cas9 gene-editing technology to be used in the treatment of germline genes, Gene editing for reproductive purposes may irreversibly change the human genome and bring incalculable impact to mankind. In the future, more evidence should be collected from animal experiments to ensure the safety and feasibility of CRISPR/Cas9 gene-editing technology in clinical practice.

The genetic complexity of non-small cell lung cancer is one of the main causes of chemotherapeutic drug resistance. Unfortunately, no effective gene-targeted drugs have been developed. We reviewed and summarized the progress of CRISPR/Cas9, which provides a reference for further research on the application of CRISPR/Cas9 gene-editing technology in the treatment and drug resistance of non-small cell lung cancer. We believe that further systematic and in-depth research is necessary. We need to make full use of the advantages of CRISPR/Cas9 gene-editing technology, explore its potential in the study of drug resistance mechanisms, promote the rapid development of cancer research and bring new hope to cancer patients.
